# The effect of sodium hypochlorite and resin cement systems on push-out bond strength of cemented fiber posts

**DOI:** 10.12669/pjms.324.10235

**Published:** 2016

**Authors:** Fahad I. Alkhudhairy, Mohammed S. Bin-Shuwaish

**Affiliations:** 1 Fahad I. Alkhudhairy, BDS, AEGD, MS, SBRD, ABOD. Assistant Professor, Department of Restorative Dental Sciences, King Saud University College of Dentistry, Riyadh, Saudi Arabia; 2 Mohammed S. Bin-Shuwaish, BDS, AEGD, MS, ABOD. Assistant Professor, Department of Restorative Dental Sciences, King Saud University College of Dentistry, Riyadh, Saudi Arabia

**Keywords:** Adhesive cements, Bond strength, Concentration, Fiber post, Push-out test, Sodium hypochlorite

## Abstract

**Objective:**

This study investigated the effect of different endodontic irrigant solutions and resin cement systems on the bond strength of cemented fiber posts.

**Methods:**

Sixty human single-rooted anterior teeth were sectioned transversely at 2 mm incisal to the cemento-enamel junction (CEJ). The roots were treated endodontically, and teeth were distributed into six groups: group A, includes 5.25%NaOCl irrigant with MultiCore Flow Core Build-Up material; group B, includes 5.25%NaOCl irrigant with RelyX-Unicem Self-Adhesive Universal Resin Cement; group C, includes 2.5% NaOCl irrigant with MultiCore Flow; group D, includes 2.5%NaOCl irrigant with RelyX-Unicem; group E, includes NaCl, irrigant with MultiCore Flow; and group F, includes NaCl irrigant with RelyX-Unicem. Universal tapered fiber posts (No. 3 RelyX Fiber Post) were cemented, and roots were sectioned into cervical and apical segments. Samples were then subjected to a push-out bond strength test and failure modes were examined.

**Results:**

The mean push-out bond strength for group D showed the highest mean value (20.07 MPa), while the lowest value was found in group A. There was a significant difference between groups with regard to the irrigants used (p<0.001), however, no significant difference was found between groups with regard to resin systems (p>0.05). The total mean push-out bond strength of the cervical segments was found to be significantly higher than the apical segments (p<0.001).

**Conclusion:**

The irrigant solution have a clear influence on the push-out bond strength of the fiber posts regardless of the cement used. Both adhesive resin systems showed similar bonding strength.

## INTRODUCTION

The restoration of endodontically treated teeth has undergone significant changes in the recent years.^1^ The demands of esthetics and strength play an important role in selecting the optimum restoration for endodontically treated anterior teeth.^2^ Currently, composite resin build up using fiber post with adhesive resin cement has become widely accepted and gaining more popularity.^3^

The histological characteristics of the root dentin following endodontic treatment in addition to the properties of the different bonding materials make the cementation of fiber posts a challenging procedure.^4^ Several factors contribute to this difficulty, such as limited access to the root canal, moisture control, and lack of direct vision during handling of the materials.^5^ The adhesion of the post to the root canal system can be affected by different elements. Effect of irrigant solutions, in varying concentrations, on dentin collagen,^6^ as well as the density and orientation of the dentin tubules in the root canal walls^7^ may have an effect on the adhesion. Moreover, the polymerization stress of the resin cement, the presence of thick smear layer, in addition to the cementation approach used and the chemical and physical properties of the posts are all contributing factors that can possibly effect the quality of adhesion at the post-cement–adhesive–dentin interfaces.^8^

Self-adhesive cements have different chemical compositions and application techniques that influence bonding performances. They were designed to adhere to the tooth structure without using a separate etchant and bonding agent. In addition, these systems are composed of conventional methacrylate monomer, acid- based monomer used to condition tooth substrate, glass particles, and initiator-accelerator system to start and complete polymerization process. They were manufactured to simplify the cementation procedures and reduce the technique sensitivity that was associated with multiple-step adhesive systems.^9^ These resin cements have a low demineralization effect, especially if the dentin is covered with thick smear layer, and it does not form a distinguishable hybrid layer.^10^ Among the different bond strength testing procedures, push-out bond strength testing is considered the most reliable for recording the bond strength failure similar to the clinical situation.^11^

The routine use of sodium hypochlorite (NaOCl) as the primary endodontic irrigant solution is justified by its wide-spectrum antimicrobial activity and its unique capability to dissolve organic tissue remnants.^12^ After irrigation to remove the smear layer, residual chemicals are likely to diffuse along dentinal tubules which may affect the penetration of resin into dentinal tubules or inhibit the polymerization process of resin cement.^13^ Consequently, this will affect the bond between adhesive systems and the treated (demineralized) dentin, and reduce the durability of the cemented post and restoration.

The aim of this study was to evaluate the effect of NaOCl and two resin systems on bond strength of cemented fiber posts, and to evaluate their failure modes.

The study hypotheses were:

1. There would be no significant difference in bond strength of the fiber posts, to root canal dentin, between two groups cemented with MultiCore Flow Core Build- Up or with self-adhesive RelyX-Unicem.
2. There would be no significant difference in bond strength of the fiber posts to root canal dentin between groups irrigated with 5.25% sodium hypochlorite; 2.5% sodium hypochlorite; or with saline

## METHODS

### Sample selection and preparation

Sixty human single-rooted maxillary permanent central and lateral incisors, with root length of more than 14 mm, were selected. Freshly extracted teeth were immediately placed in 5.25% NaOCl for 5 minutes, then stored in distilled water until further use. All teeth were evaluated under 20X magnification to exclude any teeth which displayed a crack or craze line. Teeth with caries, cervical erosion, previous endodontic treatment, post or crown were also excluded.

The crown of each tooth was sectioned at 2mm incisal to the CEJ with a diamond bur at high speed under copious water-cooling. Standardized endodontic procedure was then performed until a master apical file of 0.46 was achieved. A final flush with 2ml of 17% Ethylene-diamine-tetra-acetic acid for 20 seconds was used to irrigate the root canal. The canals were dried with sterile paper and a gutta-percha master cone coated with AH-26 sealer (Dentsply De Tray, Konstanz, Germany) was condensed into the canal.

### Grouping of specimens

The root-canal treated teeth were randomly assigned into six groups of 10 teeth each (Table-I).

**Table-I:** List of groups with the respective irrigant solution and adhesive system used in each group.

Groups (n=10 teeth)	Irrigant Solution	Adhesive System	Manufacturer of the Adhesive System
A	5.25% NaOCl	MultiCore Flow	Ivoclar Vivadent Inc, Amherst, NY, USA
B	5.25% NaOCl	RelyX-Unicem	3M ESPE, St. Paul, MN, USA
C	2.5% NaOCl	MultiCore Flow	Ivoclar Vivadent Inc
D	2.5% NaOCl	RelyX-Unicem	3M ESPE
E	NaCl	MultiCore Flow	Ivoclar Vivadent Inc
F	NaCl	RelyX-Unicem	3M ESPE

In all groups, a size # 3 tapered fiber post of 15mm length and 1.6mm diameter (3M ESPE RelyX Fiber Post, St. Paul, MN, USA) was used. To create an 11mm post space, the condensed gutta-percha was removed using a heated plugger. The post space was then irrigated with 2ml of the assigned irrigant solution. With a low speed hand piece, sequential reamers (3M ESPE) were used to shape the canals. RelyX-Unicem (3M ESPE) and MultiCore Flow (Ivoclar Vivadent Inc, Amherst, NY, USA) were used to cement the fiber posts according to the manufacturers’ guidelines.

### Push-out bond strength assessment

**Fig.1:**
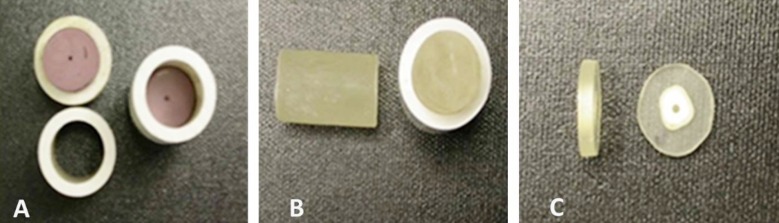
Sectioned post specimens used for the push-out test. A. Supporting PVC mounting Jig; B. Koldmount in the jig after hardening; C. The sectioned specimens for testing.

Samples were embedded in a cylindrical Polyvinyl Chloride (PVC) mounting jig to facilitate perpendicular sectioning (Fig.1a). A clear self-curing specimen mounting material “Koldmount” (Vernon-Benshoff Company, Albany, NY, USA) was mixed according to the manufacturer’s instructions and poured into the mounting ring and allowed to set for 20 minutes at room temperature (75 to 80°F) (Fig.1b).

After setting, the mounted specimen was stabilized to a metallic base in a low speed diamond saw (Model 650, SBT South Bay Technology Inc, Arlington, VA, USA), and was sectioned to cervical and apical specimens to yield twenty segments for each group (n=20) (Fig.1c).

To evaluate the effect of different irrigant solutions on bond strength, samples were subjected to a push-out test using a universal testing machine (Instron.Co, MA, USA). A metallic device, 7mm in height, 2cm in diameter, and with a central opening slightly larger than the diameter of the root canal orifice was used to hold the tooth sections. To insure the load was only concentrated on the cemented post, a 1.2mm plunger which is slightly smaller than the diameter of the cemented post was used to apply the load at a rate of 1mm/min in an apical-coronal direction until the post dislodged.

The bond strength σ (MPa) was calculated using the following formula:

σ=C/A

Where C=rupture load of each specimen (N), A=bonded area (mm^2^). The value of bonded area “A” was calculated using the equation:

A=2πrh

Where π=3.14 (constant), r=radius of post, and h=length of specimen measured by a digital caliper.

### Failure analysis

After push-out testing, the failed specimens were evaluated under a stereomicroscope (Swift-Stereo-Eighty-Microscope, Swift Instruments International, Tokyo, Japan) to determine failure modes.

### Statistical analysis

Mean and standard deviation values of bond strength were calculated. One-way analysis of variance (ANOVA) and Post-Hoc-Tukey HSD multiple comparison tests were used to analyze the differences in bond strength between the irrigant groups. Independent t-tests were also used to compare the bond strength between specimen positions for each irrigant group. Statistical software (SPSS version 20; IBM Corp) was used for the analysis and the significance level was set at α=0.05.

## RESULTS

Group D showed the highest mean push-out bond strength (20.07±5.08Mpa) among all tested groups, while the lowest values were recorded in group A (14.13±2.82Mpa), with statistical significant difference between these two groups as shown in Table-II.

**Table-II:** Push-out bond mean strength values of the different groups in Mpa (± SD).

Groups (n=20 segments)	Mean	SD	Minimum	Maximum
A	14.13^a^	2.82	10.02	19.61
B	16.27^ab^	3.26	10.36	20.78
C	19.40^b^	6.37	10.15	28.19
D	20.07^b^	5.08	12.33	28.45
E	15.37^a^	3.37	9.01	21.38
F	14.68^a^	3.20	9.76	21.97
Total	16.65	4.72	9.01	28.45

Superscript (a, b, ab) show the different significance levels. Means with different superscripts differ significantly (p<0.05), means sharing the same superscript are not significantly different.; Post Hoc tests.

The results of the one-way ANOVA revealed a highly significant difference with regard to the irrigants used (p<0.001) but no significant differences were found based on the resin system used (p=0.359) and with the irrigant and cement system combined (p=0.325). Table-III presents the ANOVA and post hoc Tukey tests analysis used to calculate the differences between groups based on the cervical and apical segments. The mean push-out bond strength in the cervical segments was found to be significant between the groups (p<0.001). The mean push-out bond strength in the root canal was significantly affected by the root region with regard to group C, and D (p<0.001), apart from group E which used saline and MultiCore (p<0.05). The independent t tests results showed that the mean push-out bond strength values of the cervical segments was significantly higher than the apical segments in the above-mentioned three groups (p<0.05). Moreover, the total mean push-out bond strength of the cervical segments (18.77±5.06Mpa) was found to significantly higher than the apical segments (14.54±3.20Mpa) (p<0.001). Multiple comparisons of the segments by post hoc tests revealed significant differences between groups only for the cervical segments with regard to group 3 and 4 (p<0.05).

**Table-III:** Push-out bond mean strength values of the cervical and apical segments in MPa (± SD).

Groups (n=20)	Cervical segment (n=10 segments each)	Apical segment (n=10 segments each)	P value
A	14.80a±3.03	13.45±2.56	0.294
B	17.37a±2.94	15.18±3.33	0.136
C	24.76b±2.62	14.04±3.88	0.000*
D	23.66b±3.86	16.48±3.30	0.000*
E	17.23a±2.87	13.51±2.82	0.009*
F	14.80a±3.69	14.56±2.82	0.870
Total	18.77±5.06	14.54±3.20	0.000*
P value	0.000*	0.258	

* Statistically significant P-value (p<0.05), Independent t-test for the segments and ANOVA for the total sample. Means with different superscripts in a column differ significantly (p<0.05), means sharing the same superscript are not significantly different.; Post Hoc tests

**Fig. 2:**
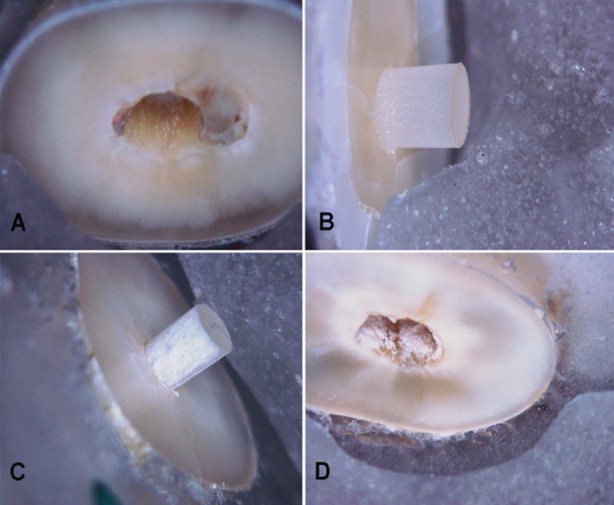
Failure modes of the push-out specimens, A. Adhesive-Dentin (cement debonded from dentin surface), B. Adhesive-Post (cement debonded from the post surface), C. Cohesive- Cement (failure within cement layer), D. Mixed failure (indicated by comparable amounts of cement adhering to the post and radicular dentin).

Most of the specimens (71.67%) failed predominately at the interface between the adhesive and the dentin surface (Fig.2a) and only two specimens (1.67%) showed cohesive failure (Fig.2c) as shown in Table-IV. Among 15% of adhesive failures from the post (Fig.2b), there were relatively more numbers observed in the group B and mixed failures (Fig.2d) were observed more in the group C.

**Table-IV:** Type of failure modes in each group.

Groups (n=20)	Root segments	Failure modes			

		Adhesive-dentin	Adhesive-post	Cohesive	Mixed
A	CERVICAL	7	0	0	3
	APICAL	9	1	0	0
B	CERVICAL	5	4	1	0
	APICAL	6	2	0	2
C	CERVICAL	5	3	0	2
	APICAL	7	0	0	3
D	CERVICAL	8	1	0	1
	APICAL	9	0	0	1
E	CERVICAL	9	1	0	0
	APICAL	6	3	1	0
F	CERVICAL	8	0	0	2
	APICAL	7	3	0	0
Total (%)		86 (71.6)	18 (15.0)	2 (1.67)	14 (11.66)

## DISCUSSION

In this study, upper central and lateral incisors were selected because they have similar root canal anatomy. The push-out strength test is not only a simple and valid method but also allows fabrication of a number of specimens out of one root, as well as testing for regional differences between root sections.^14^ Moreover, the push-out test has demonstrated a more homogenous stress distribution in finite element analysis, lower data variability and no occurrence of premature failures.^15^ However, it is difficult to compare equitably the bond strengths from different testing specimens by using the push-out test.

On comparing the bond strength of the tested two resin systems (MultiCore Flow Core Build- Up and self-adhesive RelyX-Unicem), in association with the three irrigant solutions (5.25%; 2.5% sodium hypochlorite; saline), respectively, there were no significant differences between the adhesive systems groups. This finding concurs with a previous investigation which reported that the cements performed similarly in terms of push-out bond strength^16^ and thereby supporting the first hypothesis, there is no significant difference, in bond strength of the fiber posts to root canal dentin between the two tested resin systems. However, the second hypothesis, there is no significant difference, in bond strength of the fiber posts to root canal dentin between different irrigant groups was rejected. The results of this study is in accordance to other studies that reported a higher bond strength for the cervical than apical segments.^17^,^18^

A possible explanation for the increase in adhesive values in the cervical portion of the canal is because it is the most accessible part of the canal space. However, a decrease in the adhesive values in the apical region might be related to the thickness in the distribution of resin cement with probable void formation or traces of remnant gutta-percha and endodontic sealer after post-space preparation and also dependent on the concentrations of NaOCl used. Another reason may be due to the varying densities and distribution of dentinal tubules evident in the different regions of the root canal.^19^

The impact of NaOCl concentration on bond strength is still a controversial topic. Lower bond strength with exposure to 5%NaOCl has been reported because it oxidizes some component of the dentine matrix and inhibits the polymerization of resins,^20^ while another study showed positive results in favor of an increased concentration of NaOCl until it reached a plateau at a concentration of 10% with an application time of 60 seconds.^21^ Our results have confirmed the adverse impact of the higher concentration of NaOCl on the bond strength of fiber post; hence it is advisable to use an anti-oxidant agent before post cementation.

In the post-adhesive interface, the retaining capacity relies on the mechanical physical interlocking and chemical retention if any, or as a result of friction. The other interface is the adhesive-dentin interface, which relies on various factors like dentin substrate conditioning and bonding procedures, since the dentin could be etched with self-etch AdheSE DC as with MultiCore or not etched or condition the dentin as in the case of RelyX-Unicem.

A higher number of adhesive-dentin interface failures were observed in this study. This result is in agreement with several studies that used self-adhesive cements.^22^,^23^ Firstly, this may be due to the fact that self-adhesive resin cement does not form a distinct hybrid layer between dentin and resin tags like etch-and-rinse or self-etching adhesive systems.^19^ Furthermore, the self-adhesive system is unable to etch a thick smear layer and demineralize the dentin surface.^24^ In the presence of smear layer that is not removed as with RelyX-Unicem, the dislocation resistance of bonded fiber posts was contributed largely by sliding fiction. Secondly, the residual from NaOCl may reduce the bond strength and inhibit the polymerization of resin cement. NaOCl forms a thin layer of oxygen along the entire dentin surface leading to weakness in the adhesive-dentin interface.^20^

The present study showed that irrigant solutions with different concentrations played an important role in influencing the bond strength of the fiber posts regardless of the cement used, therefore, careful selection of the irrigant is an important step during the post and core procedure. However, this study was limited to one type of fiber post, three types of irrigation protocols and two types of resin systems. The extent of the superficial changes on the root canal surfaces, the use of more challenging dentin substrate such as caries-affected or sclerotic dentin and the post surface treatments were not evaluated in this study, which needs further evaluation to confirm our results.

## CONCLUSION

Within the limitations of this study, the following can be concluded:

1. Irrigant solutions and their different concentrations significantly affect the push-out bond strength of the cemented fiber post
2. Post spaces irrigated with NaOCl solutions showed significantly stronger push-out bond than those irrigated with NaCl.
3. Both, RelyX-Unicem resin cement and MultiCore build up material showed similar bonding strength.
4. For all experimental groups combined, push-out bond strength was found to be higher in the cervical root segments than in the apical root segments.
